# Chronic obstructive pulmonary disease exacerbation episodes derived from electronic health record data validated using clinical trial data

**DOI:** 10.1002/pds.4883

**Published:** 2019-08-05

**Authors:** Matthew Sperrin, David J. Webb, Pinal Patel, Kourtney J. Davis, Susan Collier, Alexander Pate, David A. Leather, Jeanne M. Pimenta

**Affiliations:** ^1^ School of Health Sciences, Faculty of Biology, Medicine and Health University of Manchester Manchester UK; ^2^ Real‐World Data and Analytics GlaxoSmithKline plc. Harlow UK; ^3^ Clinical Statistics (Respiratory) GlaxoSmithKline plc. Uxbridge UK; ^4^ Real‐World Data and Analytics GlaxoSmithKline plc. Collegeville PA USA; ^5^ Respiratory Therapy Area Unit GlaxoSmithKline plc. Brentford UK; ^6^ Epidemiology, Global Medical GlaxoSmithKline plc. Uxbridge UK

**Keywords:** algorithms, chronic obstructive, electronic health records, pharmacoepidemiology, pulmonary disease, validation

## Abstract

**Purpose:**

To validate an algorithm for acute exacerbations of chronic obstructive pulmonary disease (AECOPD) episodes derived in an electronic health record (EHR) database, against AECOPD episodes collected in a randomized clinical trial using an electronic case report form (eCRF).

**Methods:**

We analyzed two data sources from the Salford Lung Study in COPD: trial eCRF and the Salford Integrated Record, a linked primary‐secondary routine care EHR database of all patients in Salford. For trial participants, AECOPD episodes reported in eCRF were compared with algorithmically derived moderate/severe AECOPD episodes identified in EHR. Episode characteristics (frequency, duration), sensitivity, and positive predictive value (PPV) were calculated. A match between eCRF and EHR episodes was defined as at least 1‐day overlap.

**Results:**

In the primary effectiveness analysis population (n = 2269), 3791 EHR episodes (mean [*SD*] length: 15.1 [3.59] days; range: 14‐54) and 4403 moderate/severe AECOPD eCRF episodes (mean length: 13.8 [16.20] days; range: 1‐372) were identified. eCRF episodes exceeding 28 days were usually broken up into shorter episodes in the EHR. Sensitivity was 63.6% and PPV 71.1%, where concordance was defined as at least 1‐day overlap.

**Conclusions:**

The EHR algorithm performance was acceptable, indicating that EHR‐derived AECOPD episodes may provide an efficient, valid method of data collection. Comparing EHR‐derived AECOPD episodes with those collected by eCRF resulted in slightly fewer episodes, and eCRF episodes of extreme lengths were poorly captured in EHR. Analysis of routinely collected EHR data may be reasonable when relative, rather than absolute, rates of AECOPD are relevant for stakeholders' decision making.

**Key Points**

- Robust and reproducible research based on routinely collected health data relies on transparent, validated code lists mapped to definitions for analytic data set preparation.
- Here, we validate a method of ascertaining moderate/severe COPD exacerbation episodes from electronic health record data, against data collected prospectively through an electronic case report form in the Salford Lung Study in COPD.
- Electronic health record‐derived outcomes may be particularly relevant where decision making can be informed by relative, rather than absolute exacerbation rates.

## 1 INTRODUCTION

Electronic health records (EHRs), created during health care delivery within systems optimized for providers, are increasingly used for research.^1^, ^2^ However, as with any secondary reuse of data, there are challenges to their use. A key challenge is that records of clinical episodes need to be derived from codes chosen by providers or automated by systems into a definition for the research protocol; for a single episode, there may be multiple definitions, built on one or more sets of codes, that could reasonably be used, and these may result in quite different findings in subsequent analyses.^3^ This is partly a result of variability in standards and the use of codes by clinicians in their routine use of EHR systems to manage care delivery.^4^ Therefore, it is important to have transparent, validated code lists and definitions to enable robust and reproducible research based on routinely collected health data.^5^

We specifically consider the difficulty in defining moderate/severe acute exacerbations of chronic obstructive pulmonary disease (AECOPD) episodes from EHRs. This is particularly challenging because episodes may be identified using a wide variety of codes for drug therapies, symptoms, and diagnoses and hospitalizations from primary care, and the resulting code lists may have both low sensitivity and low positive predictive value (PPV). Moreover, multiple codes observed on different days may correspond to the same AECOPD episode. Recently, Rothnie and colleagues developed a well‐discriminating algorithm to identify AECOPD episodes in EHR data in a UK setting and validated this using questionnaire data from general practitioners (GPs) or hospital discharge summaries, reviewed by respiratory physicians.^6^, ^7^ However, there are limitations to consider: it is not clear what the gold standard should be (ie, the start and end date of AECOPD episodes may not always be recorded by a health care professional); there is potential response bias,^8^ and predictive values observed in one study may not apply to all populations, in terms of generalizability across health care settings, systems, or geographies.^9^

In this short paper, we validate a method of ascertaining moderate/severe AECOPD episodes from EHR data, developed using the Salford Integrated Record EHR system, against AECOPD episode data collected through an electronic case report form (eCRF) in the Salford Lung Study in COPD (SLS COPD), a randomized clinical trial conducted in routine GP practice. Performance measures included sensitivity (percentage of true positives identified) and PPV (proportion of those identified who are identified correctly).

## 2 PATIENTS AND METHODS

### 2.1 Data sources

Two data sources were used for this study. First, we accessed data from the eCRF of SLS COPD.^10^ This was a phase 3B, randomized, controlled, point‐of‐care trial evaluating the effectiveness and safety of initiating once‐daily inhaled fluticasone furoate/vilanterol 100/25 μg compared with continuing usual care among patients with COPD. The annual rate of moderate/severe AECOPD was the primary end point. The trial took place in Salford and surrounding areas of England; patients were enrolled at their usual GP practice, and most data were collected as patients received care and prescriptions in their routine manner.^11^ It should be noted that, in SLS COPD, automated alerts triggered a manual review of each electronic medical record by the study medical staff, after which appropriate data were entered into the eCRF, which differs from standard clinical trial eCRF procedures.^12^

Second, for the same group of patients and the same timeframe, we accessed EHR data (Salford Integrated Record).^13^

### 2.2 Identification of outcome (moderate/severe AECOPD)

#### 2.2.1 SLS COPD Clinical Trial eCRF

Moderate AECOPD episodes in the trial were defined as worsening of respiratory symptoms leading to treatment with antibiotics or oral glucocorticoids (or both), while severe AECOPD episodes were defined as those requiring or prolonging hospital admission. Episodes were determined in several ways. The study required assessment of patient data at 3‐, 6‐, 9‐, and 12‐month time points. Community research nurses reviewed the primary care record ahead of the scheduled time‐points and checked for any relevant information (any AECOPD‐like symptoms, antibiotic or oral corticosteroid treatment, or hospital discharge letter). All information related to an AECOPD event was medically verified with the study investigator before being entered in the eCRF, ensuring that prescribed medication was for an AECOPD and not indicated for another cause. In addition, severe AECOPD episodes (those requiring hospitalization) were noted by the safety team during review of hospital admissions and were entered in the eCRF. If no GP or hospital contact was made, the community research nurses telephoned the patient and could thus report any self‐treated episodes or those managed outside of usual GP practice; as per process for all exacerbations, these episodes were also medically verified before being entered in the eCRF. Note that such self‐treated episodes would not be recorded in the routine EHR recording. Start dates of AECOPD episodes were the earliest date of GP visit, hospital admission, or date of onset of a patient‐reported event; similarly, end dates were either reported by the GP or hospital (or patient if not stated) or recorded as the end date of the AECOPD‐related prescription. A new AECOPD episode required a minimum of a 7‐day gap between the end of the initial prescription and the start of new symptoms or treatment (including patient reported).

#### 2.2.2 Salford integrated record EHR

For all patients within SLS COPD, we used existing algorithms to identify moderate/severe AECOPD.^6^, ^7^ The algorithms identify AECOPD episodes using a two‐stage procedure. First, an AECOPD episode on a given day was defined as any of the following events, using relevant Read Version 2 (primary care) or International Statistical Classification of Diseases and Related Health Problems 10th Revision (secondary care) codes:

- Antibiotic and oral corticosteroid prescriptions on the same day, each for a period of 5 to 14 days,
- Two different symptoms (cough, sputum, or breathlessness) with either an antibiotic or oral corticosteroid prescription,
- Lower respiratory tract infection code,
- Acute exacerbation of COPD code (within primary care),
- Evidence of AECOPD from the secondary care (hospital) discharge record (AECOPD as any of the recorded diagnoses or COPD as the primary diagnosis).

**Figure 1:**
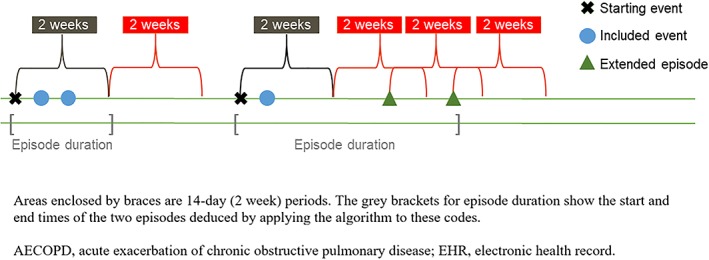
Visualization of approach to EHR‐identified AECOPD episode algorithm construction. Areas enclosed by braces are 14‐day (2‐week) periods. The grey brackets for episode duration show the start and end times of the two episodes deduced by applying the algorithm to these codes. Abbreviations: AECOPD, acute exacerbation of chronic obstructive pulmonary disease; EHR, electronic health record [Colour figure can be viewed at http://wileyonlinelibrary.com]

Second, the time spacing of the codes was analyzed to assemble events into distinct AECOPD episodes; codes nearby in time were considered to pertain to the same episode. Specifically, the start date of an episode was determined by the first relevant code and provisionally set to end 14 days later. If any further codes were identified within a 14‐day window after the provisional episode end date, the episode was extended to encompass the further code. This process was repeated until the episode end date was followed by a 14‐day period with no relevant codes (Figure 1). This approach allows for recovery time from the initial AECOPD episode,^14^ with the AECOPD‐free window beyond the end date set to distinguish between a relapse of the existing episode and a new episode.

### 2.3 Study population

The study population comprised patients enrolled in either treatment arm of SLS COPD, and we utilized the primary efficacy population (n = 2269) for this ad hoc analysis.^10^ Patient demographics have been reported in full elsewhere.^10^ In summary, the mean age of included patients was 67 years (standard deviation [*SD*] = 10 years); 49% were female; 46% were current smokers; the mean forced expiratory volume in 1 second (post‐bronchodilation) was 1.59 L (*SD* = 0.64), and the mean number of moderate/severe AECOPD in the prior 12 months was 2.5 (*SD* = 1.9). Having had at least one moderate/severe AECOPD in the prior 3 years was an inclusion criterion for SLS COPD.

### 2.4 Statistical analyses

We assessed moderate/severe AECOPD episodes using the existing algorithms in the Salford Integrated Record EHR for trial patients in both the usual care and initiation of treatment with fluticasone furoate/vilanterol arms,^6^, ^7^ comparing them with episodes reported in the eCRF for the same patients within the same time period. We compared descriptive characteristics regarding the episodes according to the two definitions including total and mean numbers of episodes and total and mean episode lengths. The main outcome measures were sensitivity and PPV. We treated moderate/severe AECOPD episodes in the eCRF data as the “gold standard” and anchored the EHR episodes against this. Following standard nomenclature, we utilized the definitions given in Table 1 for assessment of algorithm function. For sensitivity and PPV, 95% confidence intervals were calculated using Wilson's score method with continuity correction.

**Table 1:** Definitions used for analysis of algorithm function

Term	Definition
True positive	eCRF episode with a matching EHR episode, even if an EHR episode is used more than once
False positive	EHR episode with no matching eCRF episode
False negative	eCRF episode with no matching EHR episode
Sensitivity	Calculated as true positives / (true positives + false negatives)
Positive predictive value	Calculated as true positives / (true positives + false positives)

Abbreviations: eCRF, electronic case report form; EHR, electronic health record.

**Figure 2:**
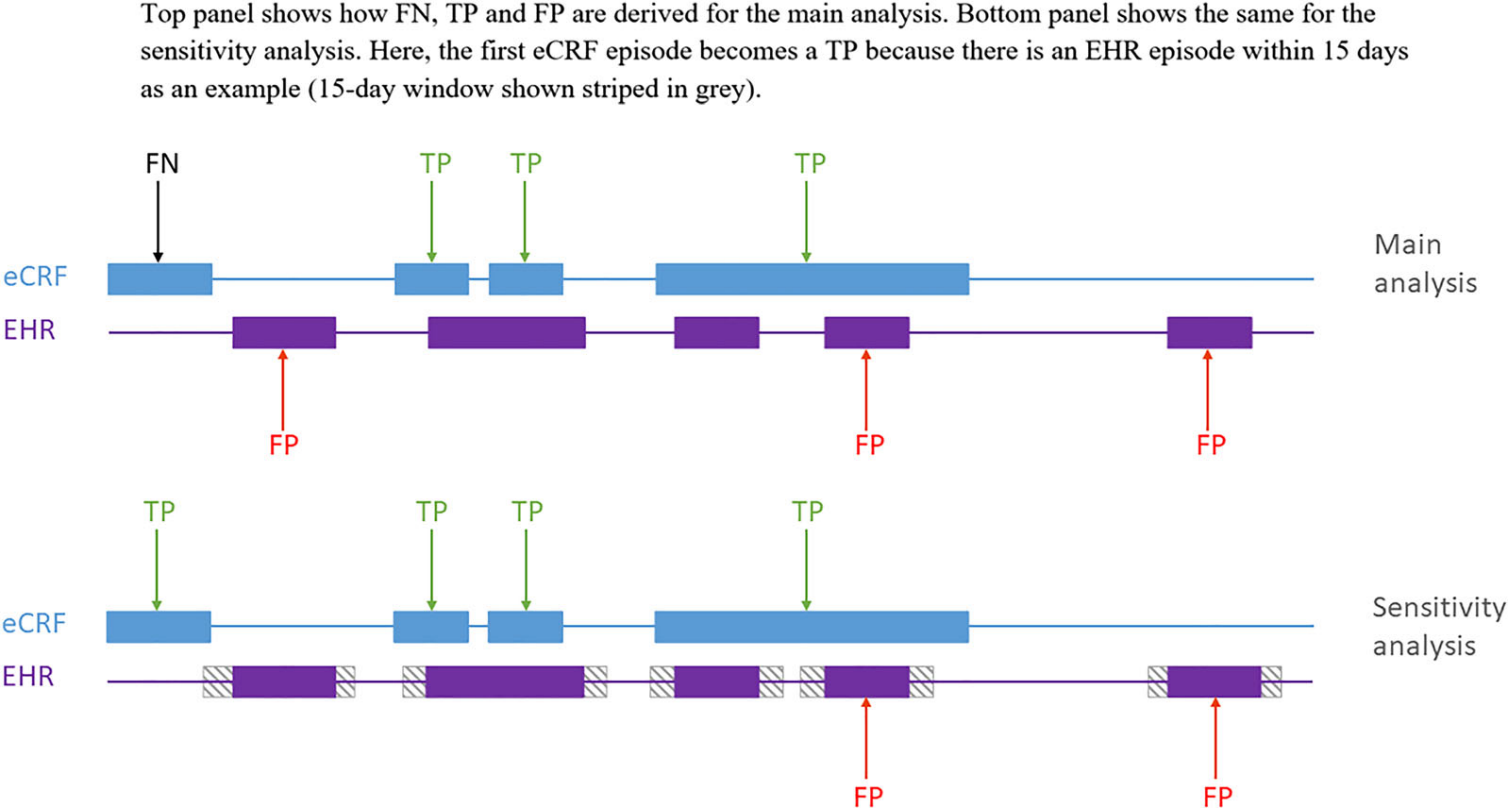
Visualization of agreement between eCRF and EHR‐derived AECOPD episodes Top panel shows how FN, TP, and FP are derived for the main analysis. Bottom panel shows the same for the sensitivity analysis. Here, the first eCRF episode becomes a TP because there is an EHR episode within 15 days as an example (15‐day window shown striped in grey). Abbreviations: AECOPD, acute exacerbation of chronic obstructive pulmonary disease; eCRF, electronic case report form; EHR, electronic health record; FN, false negative; FP, false positive, TP, true positive [Colour figure can be viewed at http://wileyonlinelibrary.com]

Determining whether an eCRF episode and an EHR episode matched depended on their proximity in time. In the primary analyses of sensitivity and PPV, we required that any portion of the episodes overlapped in time by at least 1 day to be considered a match (this equates to a distance between episodes of 0 days). In further analyses, we relaxed this definition to allow the episodes to be within up to 3, 7, 15, and 30 days of each other; for example, an EHR episode was within 3 days of an eCRF episode if any day of that EHR episode was within 3 days of any day of the eCRF episode. For each eCRF episode, and since the eCRF was considered the gold standard, one true positive was recorded for every episode with a matching EHR episode; if no match was found, the count of true negatives was incremented by one. EHR episodes that were not assigned to any eCRF episode in this way were considered false positives. During the trial, patients were contacted by research nurses every 3 months in an effort to ensure that no exacerbations were missed from the eCRF; therefore, there is a very low likelihood that an episode derived from the EHR with no corresponding episode recorded in the eCRF, was genuine. When multiple (“n”) EHR episodes covered a single eCRF episode, this was counted as one true‐positive and “n‐1” false‐positive episodes (Figure 2). This could occur if an episode involved long spells of treatment (especially self‐treatment), which would mean a single episode would be recorded in the eCRF, but long time gaps between codes recorded in EHR are translated into a series of separate episodes.

Furthermore, to assess the relative benefits of utilizing either primary care only or linked primary and secondary care, we considered two versions of the algorithm for comparison to the eCRF: (a) combining AECOPD codes, antibiotic, and oral corticosteroid codes and lower respiratory tract infection codes and (b) repeating the first algorithm with the addition of hospital admissions for COPD.

All analyses were conducted using SAS version 9.4, and results were quality assured by a second data analyst who performed independent programming.

## 3 RESULTS

The primary effectiveness analysis population comprised 2269 patients. A total of 4423 AECOPD episodes were identified using eCRF data; 20 of these episodes were excluded from the analyses as the start or end dates were unknown (n = 4403). Overall, 3791 AECOPD episodes were identified when primary and secondary care EHR data were used, while 3577 AECOPD episodes were identified when primary care only EHR data were used.

**Figure 3:**
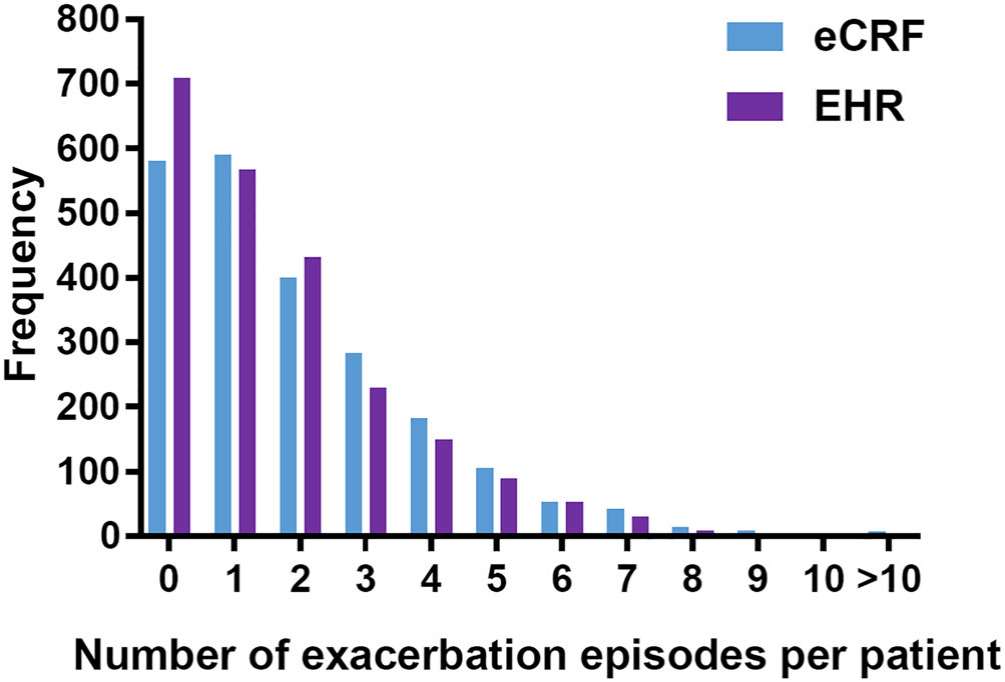
Frequency of AECOPD episodes per patient in eCRF compared with EHR (primary and secondary care). Abbreviations: AECOPD, acute exacerbation of chronic obstructive pulmonary disease; eCRF, electronic case report form; EHR, electronic health record [Colour figure can be viewed at http://wileyonlinelibrary.com]

**Figure 4:**
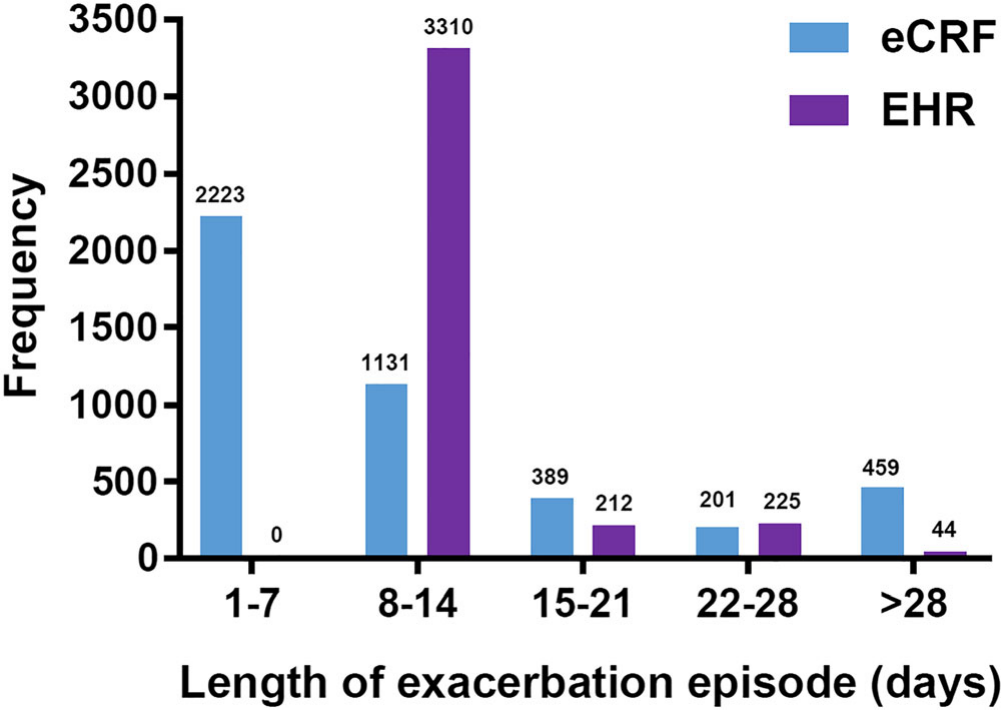
Length of AECOPD episodes in eCRF compared with EHR (primary and secondary care). Abbreviations: AECOPD, acute exacerbation of chronic obstructive pulmonary disease; eCRF, electronic case report form; EHR, electronic health record. [Colour figure can be viewed at http://wileyonlinelibrary.com]

The median number of AECOPD episodes per patient was one, irrespective of the method of identification (eCRF or EHR). The mean (*SD*) number of episodes using eCRF was 1.9 (1.96) and for EHR was 1.7 (1.74), using primary and secondary care data (Figure 3). Using primary care only EHR data, the mean (*SD*) number of episodes was 1.6 (1.70). The mean (*SD*) length of eCRF‐identified AECOPD episodes was 13.8 days (16.20), whereas the mean duration of EHR‐identified AECOPD episodes using primary and secondary care data was 15.1 days (3.59) (Figure 4). EHR‐identified AECOPD episodes using primary care only data had a mean duration of 15.0 days (3.36). Longer eCRF episodes (in excess of 28 days) were usually broken up into separate, shorter episodes in the EHR, because of long gaps between codes being recorded.

In initial analyses to determine whether an eCRF episode and an EHR episode matched, concordance was defined as an overlap of at least 1 day (equivalent to a distance or gap of 0 days) between AECOPD episodes. When primary and secondary care EHR data were included, 2801 eCRF‐identified AECOPD episodes were also identified in the EHR. Thus, of the 4403 eCRF‐identified episodes overall, 1602 episodes were not identified in the EHR; conversely, 990 EHR‐identified AECOPD episodes were not reported as eCRF‐identified episodes. This resulted in a sensitivity of 63.6% and a PPV of 71.1% (Table 2). In further analyses, allowing longer time gaps between the matched episodes increased the sensitivity without decreasing the PPV, up to a distance between AECOPD episodes of 15 days (sensitivity = 69.1%, PPV = 73.6%); however, the PPV decreased when the time gap was extended to 30 days (sensitivity = 73.7%, PPV = 67.6%) (Table 2).

**Table 2:** Sensitivity and PPV of varying algorithm with different levels of overlap with eCRF‐identified AECOPD episodes compared with AECOPD episodes in primary and secondary care EHR data and primary care only EHR data

	Distance between AECOPD Episodes, d	AECOPD Episodes Concurrent with eCRF Episodes, n	Sensitivity, %	95% CI	PPV, %	95% CI
Primary and secondary care EHR data	0^a^	2801	63.6	62.2‐65.0	71.1	69.7‐72.5
	3	2851	64.8	63.3‐66.2	72.1	70.7‐73.5
	7	2918	66.3	64.9‐67.7	73.1	71.7‐74.4
	15	3042	69.1	67.7‐70.5	73.6	72.2‐74.9
	30	3245	73.7	72.4‐75.0	67.6	66.3‐68.9
Primary care only EHR data	0^a^	2690	61.1	59.6‐62.5	72.4	70.9‐73.8
	3	2746	62.4	60.9‐63.8	73.6	72.1‐75.0
	7	2807	63.8	62.3‐65.2	74.5	73.1‐75.9
	15	2932	66.6	65.2‐68.0	75.1	73.8‐76.5
	30	3138	71.3	69.9‐72.6	69.1	67.7‐70.4

Abbreviations: AECOPD, acute exacerbation of chronic obstructive pulmonary disease; CI, confidence interval; eCRF, electronic case report form; EHR, electronic health record; PPV, positive predictive value.

a Zero days distance between episodes is equivalent to 1‐day overlap.

Repeating these analyses using primary care only EHR data, initial analyses (concordance, 1‐day overlap) resulted in 2690 AECOPD episodes that were concurrently identified by eCRF and EHR, with a sensitivity of 61.1% and PPV of 72.4%. Following the same trend as the analysis of linked primary and secondary care data, when primary care only data were used, the sensitivity increased, in this case by approximately 10%, as the gap between AECOPD episodes was increased to 30 days (sensitivity = 71.3%, PPV = 69.1%) (Table 2).

## 4 DISCUSSION

We have compared AECOPD episodes derived algorithmically from a routinely collected EHR with the episodes recorded in a clinical trial eCRF from a corresponding population. Fewer episodes were identified from the EHR compared with the eCRF (3791 vs 4403), suggesting that any focus of evidence generation using only EHR‐derived episodes should be on relative rather than absolute rates. Moreover, not all episodes identified in routine EHR matched those from the eCRF. In the best‐case scenario, using the full algorithm in primary and secondary care, and allowing a maximum gap in the start or end dates of the episodes of up to 15 days, we calculated sensitivity of 69.1% and maximal PPV of 73.6%. Whether these sensitivity and PPV figures are deemed sufficient would depend on the specifics of the study question and the type(s) of decision making impacted by the results.

The original validation study of the AECOPD episode algorithm used questionnaire data from GPs as the gold standard, compared with routine EHR data collection.^6^ For their full algorithm excluding hospital admission, the study yielded a sensitivity of 62.9% and PPV of 85.5% (vs our figures of 61.1% and 72.4%). Thus, we observed similar sensitivity but slightly lower PPV; the lower PPV may reflect differences in study design and the population included.

The median duration of an AECOPD episode was defined as 7 days in the eCRF and 14 days in the EHR algorithm. Of note, additional information was available for the eCRF (patient‐reported symptoms and treatments), allowing potentially longer episodes to be constructed based on this information that may appear temporally between coded information. This difference in data captured by source had a strong impact on the distributions of episode lengths: eCRF episodes were shorter on average but had a higher *SD*. Thus, health care utilization calculations per episode will be higher, and perhaps overestimated, in the case of the EHR algorithm. Additionally, some patients had access to rescue oral steroid packs to self‐medicate in the event of worsening COPD; this may have reduced the length of their AECOPD episodes, which would have been recorded in eCRF data, through patient reporting to the study nurse, but not in routinely collected EHR data.

More generally, there is limited literature concerning the validation of outcomes from EHRs against outcomes recorded in an eCRF during a clinical trial. One recent study compared researcher‐measured vs EHR‐derived weight in weight loss trials, finding high agreement between these measures.^15^ However, weight is a single measure and therefore a far simpler scenario than we have considered in the complex definition of moderate/severe AECOPD episodes. An ongoing phase IV trial is comparing EHR‐ and eCRF‐recorded major adverse cardiovascular events in type 2 diabetes; the agreement between these measures will be interesting to consider in the context of our own findings in COPD.^16^ Quality of data captured by both eCRF and EHR will depend on the overall system of data collection and quality assurance, as well as incentives to record information accurately.

This study has limitations. First, where an episode is recorded in both the eCRF and EHR at similar times, it can be challenging to infer whether they correspond to the same episode. For example, the date of onset of an eCRF exacerbation was based in some cases on a patient report of symptom onset; however, it may take several days before a patient is able to see their GP, which would lead to the later date being recorded in the EHR for the same exacerbation. To overcome this lag time, we considered a range of gap times between the reporting in the two sources and found that the results were relatively stable to the choice of gap. Second, the EHR algorithm was originally derived from UK data, and here has been validated using UK data. Hence, the algorithm may need modifying for application in other countries with different health care delivery, recording practices, and incentives and may not perform as well. Third, the setting for this study was a randomized clinical trial at the point of care (GP practice). As the increased awareness and monitoring (potential for a Hawthorne effect) in the trial is likely to have improved recording in the EHR during the course of the trial, we might expect, outside of this context, more difficulty in inferring AECOPD episodes from EHRs. Specifically, in related work, when comparing these EHR data with EHR data from the rest of the United Kingdom, we found that coding rates for AECOPD were high (98th percentile).^17^ However, the setting did allow us a unique opportunity to validate the algorithm against a different data collection method (eCRF). Finally, we acknowledge that, as it is common when validating EHR algorithms, we did not have a robust gold standard as a comparator. Here, we used episodes reported in the eCRF for a clinical trial, while an original validation used questionnaires sent to GPs; however, these are all approximations of the ground truth.^18^ The ideal method of validating the EHR data is likely to be a combination of detailed case note review with input from the GPs, and consideration of patient‐reported information, particularly related to the timing of self‐managed acute exacerbations. Furthermore, the external validity of the approach should be examined by repeating the approach in at least one other trial with concurrent EHR data available.

To date, regulatory decision making based on real‐world evidence typically focuses on post‐marketing safety evaluations (eg, observational postmarketing and postauthorization safety studies and registries).^19^ Outside of the context of rare diseases and oncology, however, adoption of real‐world effectiveness data to inform labelling has been hampered by concerns about data quality and accuracy, choice of comparators, and limitations of the methods used to adjust for potential confounding by severity/indication. Maximizing the use of opportunities to validate EHR data against clinical trial data, as we have done here, may help address some of the quality concerns raised for utilizing real‐world evidence to inform decision making beyond safety‐related outcomes.

A pragmatic trial design comparing a new medication to usual care, which includes data collection based on EHR data, can allow comparative effectiveness to be demonstrated in wider populations than traditional more restricted trial designs, producing results that are more generalizable to inform decision making.^20^ Pragmatic designs that rely in part or in whole on EHR data need to consider trade‐offs in efficiency and relevance with differences in data quality and completeness compared with eCRF‐based data collection.^21^ However, more recent advances in improving real‐world data capture within routine systems, such as modifications to EHR systems, incentives for improved quality assurance, and increased training for health care professionals to participate in clinical research, as well as linkage to remote data capture by patients/devices, and advances in analytic tools/methods, are increasing the infrastructure to enable robust evidence generation that can inform important regulatory decision making beyond questions of postmarketing safety.

In conclusion, we have shown that, in a real‐world point‐of‐care randomized trial context, EHR‐derived outcomes may offer an acceptable and more efficient alternative to resource intensive eCRF‐derived endpoints, particularly where relative, rather than absolute, rates are pertinent as fit‐for‐purpose evidence to inform stakeholders' decision making. The development of real‐world data standards, transparent algorithms for key outcomes, and implementation to harmonize EHR systems will further enable this evolution toward efficient, impactful, EHR‐enabled evidence generation.

## ETHICS STATEMENT

This study is based in part on data from the Clinical Practice Research Datalink obtained under licence from the UK Medicines and Healthcare products Regulatory Agency. The data are provided by the patients and collected by the NHS as part of their care and support. The Office for National Statistics (ONS) is the provider of the ONS data contained within the CPRD data. Hospital Episode data and the ONS data (2014) are reused with the permission of the Health and Social Care Information Centre. All rights reserved. The study is in part based on data from the Salford Lung Study: COPD (SLS COPD). All patients provided written informed consent to participate in SLS COPD, which was conducted in accordance with the International Conference on Harmonisation, Good Clinical Practice and the Declaration of Helsinki 2008. The SLS COPD study was approved by the National Research Ethics Service Committee North West, Greater Manchester South.

## FUNDING INFORMATION

This work was funded by GlaxoSmithKline plc. (study number, HZC115151; clinical http://trials.gov identifier, NCT01551758).

## CONFLICT OF INTERESTS

David J. Webb, Pintal Patel, Susan Collier, David A. Leather, and Jeanne M. Pimenta are employee of GlaxoSmithKline plc. and hold stocks/shares in GlaxoSmithKline plc.; Kourtney J. Davis was a former employee of GlaxoSmithKline plc. (employee of GlaxoSmithKline plc. at time of writing) and is a current employee of Janssen/J&J. Matthew Sperrin and Alexander Pate declare no conflict of interest.

## DATA SHARING

Anonymized individual participant data from this study plus the annotated case report form, protocol, reporting and analysis plan, data set specifications, raw data set, analysis‐ready data set, and clinical study report are available for research proposals approved by an independent review committee. Proposals should be submitted to http://www.clinicalstudydatarequest.com. A data access agreement will be required.
